# Intravesical Infection Stones Caused by Magnetic Beads

**DOI:** 10.7759/cureus.47422

**Published:** 2023-10-21

**Authors:** Issei Suzuki, Tomoya Mizuno, Tsunehito Kambara

**Affiliations:** 1 Urology, Nasu Red Cross Hospital, Otawara, JPN

**Keywords:** calculi, infected stone, bladder, magnetic beads, intravesical foreign body

## Abstract

The presence of an intravesical foreign body is a rare condition concerning the diseases of the urinary system. Intravesical foreign bodies may cause hematuria and bladder perforation, which are difficult to diagnose and treat. There are various types of foreign bodies in the bladder; however, magnetic beads are rare. Multiple beads may be inserted into the urethra. A few small magnetic beads may be removed from the urethra, but an open removal is an option for many or large beads because of calculus adherence. This case reports the successful open removal of 57 magnetic beads with calculus adherence.

## Introduction

Intravesical foreign bodies are uncommon in urological diseases. Bladder perforation due to a bladder foreign body is even rarer, but it does exist and may lead to open surgery. [1]. Foreign bodies can be of various types, including pencils, thermometers, glass rods, or toothbrushes. Another example is the insertion of magnetic beads into the bladder [2]. In the past, the largest reported size of bladder stones caused by magnetic beads was 2.5 cm. However, there have been no reports of calculi adhering to magnetic beads [2].

## Case presentation

A 52-year-old man developed a gradual onset of lower abdominal discomfort. He presented to the urology department 3 months after onset. He also complained of recurring macroscopic hematuria recently. He reported that these symptoms worsened as physical activity increased. He did not complain of storage symptoms such as overactive bladder and nocturia and voiding symptoms caused by benign prostatic hyperplasia. His medical history included gastric ulcer but no history of treatment for psychiatric disease. His only regular medication was a proton pump inhibitor, and he had no history of prescribing medications for psychiatric disease or taking illegal drugs. Digital rectal examination showed that the prostate gland was surface smooth and elastic soft; that is, the prostate gland had no tenderness suggestive of prostatitis and no induration suspicious of prostate cancer. The urine test showed occult blood and turbid urine (Table 1).

**Table 1:** Urine analysis on admission pH: potential hydrogen, WBC: white blood cell, RBC: red blood cell, HPF: high-power field

Urine analysis	Collected on admission
Color	Dark orange
Turbidity (reference: clear)	Turbid
pH	7.5
Specific gravity	1.009
Protein (reference: negative)	Negative
Glucose (reference: negative)	Negative
Ketones (reference: negative)	Negative
Blood (reference: negative)	Large
Leukocytes (reference: negative)	Large
WBC (reference: <10/HPF)	50-99/HPF
RBC (reference: <10/HPF)	>100/HPF
Nitrite (reference: negative)	Negative
Bacteria	Large
Bilirubin (reference: negative)	Negative
Casts	Negative
Epithelial cells (reference: <10/HPF)	0-1/HPF

Blood and biochemical tests showed no evidence of an elevated inflammatory response suggestive of infection, but urinalysis revealed occult blood and pyuria.

**Figure 1:**
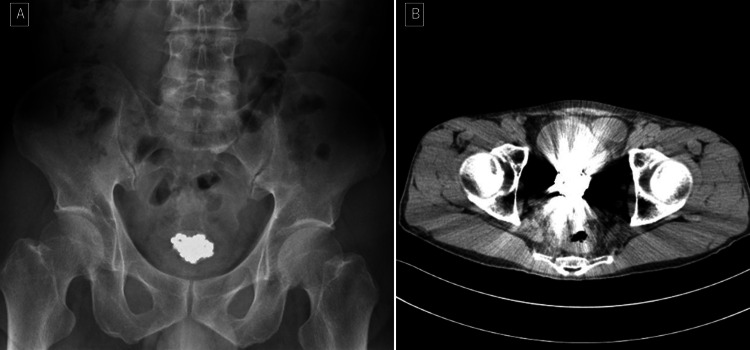
Bladder foreign body on abdominal X-ray examination (A) and on abdominal CT scan (B).

Bacteriuria was present in urine samples. Abdominal radiography showed an irregular oval body measuring 4.0 × 3.0 cm. Computed tomography revealed a high-density signal with artifacts in the bladder (Figure 1A-1B), suggesting metal properties.

**Figure 2:**
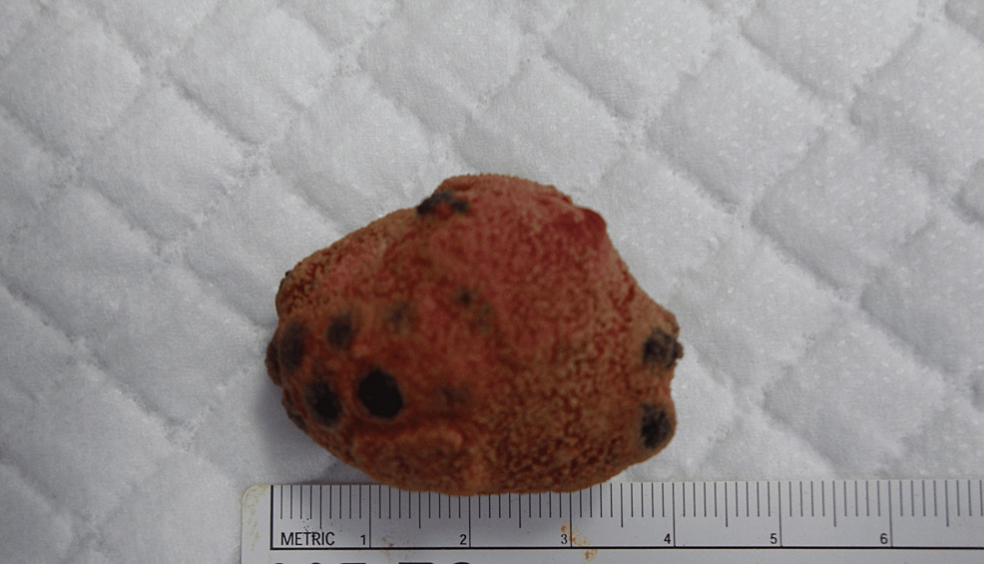
A stone attached to a magnetic bead removed from the bladder.

**Figure 3:**
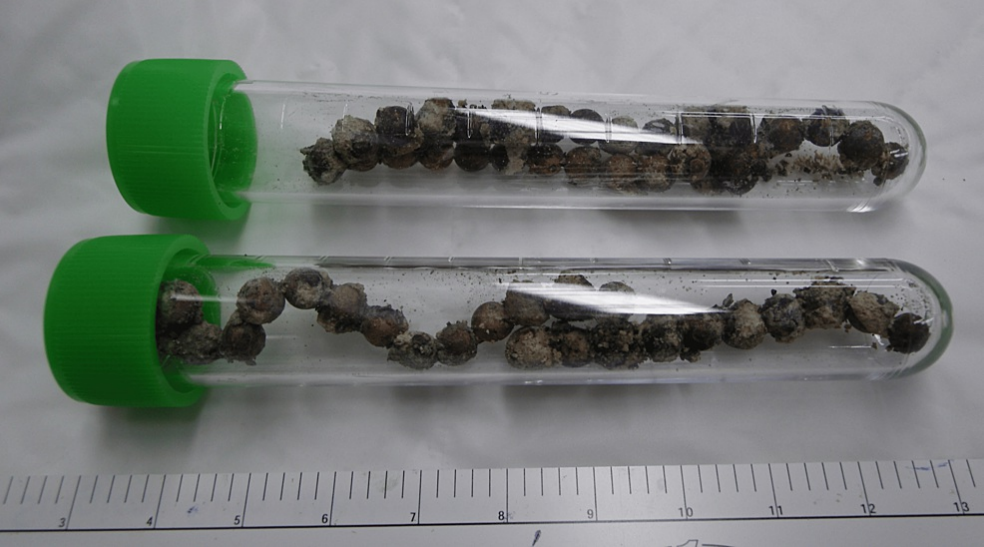
Fifty-seven magnetic beads isolated from bladder stones.

Cystoscopy revealed a large mobile stone. No fistulas were observed in the bladder. We suspected a stone containing a metal foreign body, and the patient had no memory of the insertion of the foreign body. He did not have a specific partner. He told us that about two years ago, a woman he had intercourse with had something inserted into his urethra. The details of what was inserted into the urethra at that time were not known. Because the details of the foreign body in the bladder were unknown, there were stones surrounding it, and it was large, we thought that removing the stone safely transurethrally was difficult. So, we performed an open removal procedure. We made a Pfannenstiel incision approximately 3 cm long in the lower abdomen and made a similar incision in the bladder to safely remove the foreign body. The stone contained 57 magnetic beads, having 40 g weight (Figure 2, 3).

The calculus analysis presented ammonium phosphate (83%) and ammonium acid urate (17%). The postoperative status of the patient was in good medical condition.

## Discussion

There are a few reports on using magnetic beads as intravesical foreign bodies [2]. Treatment was selected according to the foreign body's size, shape, and material. It is often removed endoscopically [3]. There are reports of the transurethral removal of magnetic beads individually [2]. Most intravesical foreign bodies can be removed transurethrally, with minimal access. All the patients reported successful management using both minimally invasive and open approaches [4].

Transurethral evacuation of large, complex, and intravesical foreign bodies is difficult and requires open removal [3]. In this case, the CT attenuation of the intravesical foreign body with calculus was high, and it seemed to be metal; however, it was difficult to determine the details. The calcified foreign body was large, and its identity of the foreign body was unknown, making it difficult to remove it from transurethrally. Therefore, open removal was selected because the magnetic beads with calcification size was 2.5 cm.

In a report summarizing urethral bladder foreign bodies caused by magnetic beads, the age ranged from 16 to 50 years, the number of magnetic beads ranged from 25 to 159, and multiple beads were inserted in all cases. Endoscopic removal was performed in seven of the 14 cases, and open removal was performed in the other cases [4]. This study does not mention calculus adhesion to intravesical foreign bodies; therefore, the details are unknown. The results of the calculus analysis were unclear [3,5]. In some studies, the details are unknown, probably because no calculus analysis was performed.

In this case, the adhering calculus was an ammonium phosphate calculus found in infected urine. The following mechanisms have been reported for calculi caused by infected urine: A biofilm layer is formed by the bacteria, and the urease breaks down urea to produce ammonia. The infected stones are composed of struvite and carbonate apatite. The basic precondition for forming infectious stones is a urease-positive urinary tract infection. Urease is necessary for splitting urea into ammonia and carbon dioxide. As a result, ammonium ions may form, and at the same time, alkaline urine develops due to the ammonia bonding with hydrogen ions to form the ammonium radical, both of which are preconditions for the formation of struvite and carbonate apatite crystals. When these crystals are deposited, infectious stones are formed [6]. This case also had a chronic infection due to the long-term placement of magnetic beads, and it was thought that infected stones caused by *Klebsiella pneumoniae*, a urease-producing bacterium, were attached around the magnetic beads in the bladder.

When a bladder foreign body is retained for a prolonged period of time, stones adhere to the nucleus of the foreign body, usually forming an infected stone. As the size of the adherent stones increases over time, transurethral endoscopic removal becomes difficult, and open removal may be necessary. It is important not to assume that bladder stones are simple, but rather to perform several imaging evaluations and carefully plan a treatment strategy.

## Conclusions

Long-term placement of magnetic beads may cause infected stones, which may grow in size over time, making endoscopic removal difficult; open removal may be needed. 
